# Family and Caregiver Characteristics Contribute to Caregiver Change in Use of Strategies and Growth in Child Spoken Language in a Parent-Implemented Language Intervention in Fragile X Syndrome

**DOI:** 10.1044/2022_persp-22-00016

**Published:** 2022-12-14

**Authors:** Sarah Nelson Potter, Lauren Bullard, Amy Banasik, Robyn Tempero Feigles, Vivian Nguyen, Andrea McDuffie, Angela John Thurman, Randi Hagerman, Leonard Abbeduto

**Affiliations:** a MIND Institute, UC Davis Health, Sacramento, CA; b Department of Psychiatry and Behavioral Sciences, UC Davis Health, Sacramento, CA; c Department of Pediatrics, UC Davis Health, Sacramento, CA

## Abstract

**Purpose:**

This study examined relationships among family characteristics, caregiver change in use of strategies, and child growth in spoken language over the course of a parent-implemented language intervention (PILI) that was developed to address some of the challenges associated with the fragile X syndrome (FXS) phenotype.

**Method:**

Participants were 43 parent–child dyads from two different PILI studies, both of which taught parents various language facilitation strategies to support child language. Before starting the intervention, parents reported on their mental health, parenting stress, and parenting competence. This study focused on potential barriers to treatment gains by examining correlations between the measures of parent well-being and (a) parent change in use of intervention strategies taught in the PILI and (b) changes in child language outcomes from preto post-intervention.

**Results:**

Parents in this study had elevated mental health symptoms across several domains and increased rates of parenting stress. Furthermore, although PILI resulted in treatment gains for both parents and children, a variety of parent mental health symptoms were found to be significantly and negatively associated with change in use of strategies and growth in child language over the course of the intervention. Some inconsistent findings also emerged regarding the relationships between parenting stress and competence and change in parent strategy use and growth in child language.

**Conclusions:**

This study provides preliminary evidence that parents who are experiencing significant mental health challenges may have a more difficult time participating fully in PILIs and that there may be subsequent effects on child outcomes. Future PILIs could benefit from addressing parent well-being as a substantial part of the intervention program.

Fragile X syndrome (FXS) is an X-linked disorder caused by an expansion of a cytosine–guanine–guanine (CGG) trinucleotide sequence on the *FMR1* gene to more than 200 repeats (Oostra & Willemsen, 2003). Individuals with more than 200 CGG repeats have the full mutation (i.e., FXS), whereas individuals with 55–200 CGG repeats are *FMR1* premutation carriers. Given the X-linked nature of FXS, males tend to be affected more often and more severely than females. Specifically, it is estimated that approximately one in 7,000 males are affected by FXS compared with approximately one in 11,000 females (Hunter et al., 2014). Moreover, the estimated prevalence of the *FMR1* premutation is one in 148 females based on population-based sample estimates (Maenner et al., 2013).

FXS is the leading inherited cause of intellectual disability (ID; Crawford et al., 2001), and more than 90% of males with FXS have IQ scores in the range of ID (i.e., < 70; Hessl et al., 2009). Intellectual functioning is more variable in females with FXS, with most females demonstrating IQs in the range of average to slightly below average intelligence (Bartholomay et al., 2019). In addition to cognitive challenges, most children with FXS experience significant delays in multiple domains of language (Abbeduto et al., 2007), with some domains affected to an even greater extent than would be expected based upon their level of cognitive functioning (Finestack & Abbeduto, 2010). Individuals with FXS may also have symptoms of autism spectrum disorder (ASD; Abbeduto, Thurman, et al., 2019; Thurman et al., 2015) and attention-deficit/hyperactivity disorder (ADHD; Chromik et al., 2019), heightened levels of anxiety (Cordeiro et al., 2011), and increased rates of challenging behaviors (Hatton et al., 2002; Oakes et al., 2016). These phenotypic challenges make engaging in interactions with affected individuals to promote the development of cognitive, language, and social skills more challenging.

## Parent-Implemented Language Interventions for FXS

To address some of the significant challenges associated with the FXS phenotype, McDuffie, Abbeduto, and colleagues developed and evaluated the efficacy of a parent-implemented language intervention (PILI) to improve spoken language in school-aged children and adolescents with FXS (Bullard et al., 2017; McDuffie et al., 2018; McDuffie, Machalicek, et al., 2016; Thurman et al., 2020). The PILI was designed to teach parents to use strategies that support their children’s language development (e.g., asking open-ended questions, such as “Where is the boy going?”) and was delivered entirely via telehealth. Delivering interventions via telehealth provides families with greater flexibility and reduces the burden of travel on families (Abbeduto, Bullard, et al., 2019). The PILI context was shared storytelling using wordless picture books that provided opportunities for the parent and child to sustain back-and-forth conversations around a shared topic.

The training for parents began with didactics in which the rationale for the intervention and targeted strategies were provided. Parents also received real-time coaching, through telehealth, and feedback, again through telehealth, on recorded homework interactions in which the parents worked with their sons. Data on parent progress were regularly assessed through telehealth and without benefit of clinician involvement.

To evaluate the efficacy of the PILI, parent–child dyads completed three shared storytelling interactions at the end of training. These interactions were recorded via secure distance teleconferencing and subsequently transcribed and coded to examine changes in parent and child performance. Across several studies, the PILI led to significant improvements in child language performance in terms of lexical diversity (i.e., number of different words) and overall relevant talkativeness (i.e., use of story-related comments). Unexpected gains in child use of inferential language were also found as a result of participation in the PILI (Nelson et al., 2018). Additionally, in these studies, parents increased their use of the three language facilitation strategies taught, as well as story-related talking, from pre- to post-intervention (Bullard et al., 2017; McDuffie et al., 2018; McDuffie, Machalicek, et al., 2016; Thurman et al., 2020). It was also found that the administration of the drug lovastatin to the youth with FXS, which was hypothesized to improve the neural pathways underlying the learning challenges associated with FXS (Osterweil et al., 2013), did not increase the benefits observed in parent strategy use or child language relative to the parent–child dyads in which the youth received a placebo (Thurman et al., 2020).

More generally, parent-implemented interventions are attractive for several reasons (McDuffie et al., 2013; McDuffie, Oakes, et al., 2016). As parents learn targeted strategies, they are likely to use them not only in the context of the intervention but also in other daily interactions (Hemmeter & Kaiser, 1994; Meadan et al., 2016). Increased use of targeted strategies by the parent provides the child with an enriched language environment compared with clinician-implemented interventions alone (Casagrande & Ingersoll, 2017; Kaiser & Roberts, 2013), and PILIs that are delivered via telehealth in the family’s home are occurring in the child’s natural learning environment. Therefore, both parent and child gains may be more likely to generalize to other contexts (Kashinath et al., 2006; Peterson, 2009). Additionally, for relatively rare disorders, such as FXS, providing PILI via telehealth allows families from rural and/or underserved communities to receive services than may otherwise be inaccessible to them (Abbeduto, 2020; Abbeduto, Bullard, et al., 2019; Hall et al., 2020).

Parent-implemented interventions also have some limitations. They are time intensive, thereby potentially placing a considerable burden on the parent who is taking on the role of the clinician (Pickard et al., 2016; Ruppert et al., 2016). This burden may exacerbate the stress that parents already face in relation to their child’s challenges. In fact, parents who experience elevated levels of mental health challenges and stress have been found to engage in less frequent and less responsive interactions with their children (e.g., Warren & Brady, 2007). Therefore, more research is needed to understand how parent well-being influences child, parent, and family outcomes for families who participate in parent-implemented interventions, and this research is particularly important in families of children with FXS where both mothers and fathers are likely to experience elevated levels of mental health challenges and parenting stress (Potter et al., 2022). In order to promote more optimal outcomes for both children and parents, future parent-implemented interventions would benefit from including goals related to parent and family functioning (Stahmer & Pellecchia, 2015).

## Parenting a Child With FXS

Parents of children with FXS typically experience elevated levels of stress given the challenges associated with raising a child (or multiple children) with significant developmental delays (e.g., Abbeduto et al., 2004; Bullard et al., 2021; Hartley et al., 2012; McCarthy et al., 2006). Moreover, biological mothers of children with FXS, who are carriers of the *FMR1* gene premutation or full mutation (i.e., FXS), have an increased genetic risk for experiencing mental health challenges above and beyond the stress associated with parenting a child with FXS (e.g., Gossett et al., 2016; Hagerman et al., 2018; Johnston et al., 2003), as well as other cognitive and medical challenges (Wheeler et al., 2014). A recent study by Potter et al. (2022) found that parenting stress was associated with both child challenging behaviors and adaptive functioning for both mothers and fathers of young boys with FXS. Therefore, families of young children with FXS would likely benefit not only from services focused on improving child outcomes but also from those focused on reducing parent stress, such as Mindfulness-Based Stress Reduction (MBSR).

The cumulative effects of the factors affecting these parents, particularly biological mothers of children with FXS, may constrain the development of warm and responsive parent–child relationships. In fact, parent mental health challenges and stress have been found to be associated with lower levels of responsiveness in interactions with the child (e.g., Sterling et al., 2013; Wheeler et al., 2007). For example, Wheeler et al. (2007) found that maternal stress was a significant predictor of the total number of maternal behaviors (i.e., maintaining and directing behaviors) exhibited during mother–child interactions, demonstrating that mothers with higher levels of stress engaged in fewer interactions with their child. Ultimately, these challenges may influence the parent’s ability to successfully learn and implement intervention strategies during interactions with their child (Casagrande & Ingersoll, 2017). Therefore, identifying ways to support parents of children with FXS who participate in parent-implemented interventions is likely to lead to improved outcomes for the family system (e.g., Bullard et al., 2021; Stahmer & Pellecchia, 2015).

## This Study

This study examined relationships among family characteristics, caregiver change in use of strategies, and child growth in spoken language over the course of a PILI delivered remotely across four different treatment conditions across two studies (McDuffie et al., 2018; Thurman et al., 2020): no PILI, PILI only, PILI + placebo, and PILI + a medication (i.e., lovastatin). In the PILI, parents learned to implement three language facilitation strategies during interactions with their child: (a) open-ended *wh*-questions (“Where is the boy going?”); (b) intonation prompts (i.e., fill-in-the-blank statements; “Then she ran to the _____.”); and (c) expansions of child verbal utterances (Child: “Happy.” Parent: “The girl is happy.”). Parents were also taught to provide the child with rich models of story-related vocabulary and grammar. Families chose 12 books that were uploaded to an iPad to use during the intervention, and, each week, a new book was used for that week’s intervention activities.

The PILI began with two parent education sessions, which introduced the parent to the rationale for and logistics of the intervention, as well as the parent language facilitation strategies. Following the parent education sessions, dyads participated in four weekly sessions via telehealth: (a) clinician coaching sessions, during which the clinician provided the parent with models, prompts, and reinforcement as they interacted with their child; (b) homework, during which the parent and child independently recorded themselves completing the shared storytelling activity together and uploaded the video for the clinician to review; (c) clinician feedback with the parent, during which the clinician and parent discussed the homework session, and the clinician provided detailed feedback regarding strategy use; and (d) data collection, during which the parent and child completed the week’s shared book reading activity for a final time without any intervention by the clinician. Overall, the time commitment for families was approximately 3 hr of initial training prior to beginning the weekly intervention sessions and 2 hr per week to complete the coaching, homework, feedback, and data collection sessions (McDuffie et al., 2018; McDuffie, Machalicek, et al., 2016; Thurman et al., 2020). Parent and child intervention-related gains were evaluated by comparing shared storytelling interactions that were collected during the 2 weeks prior to, and following, the 12-week intervention.

In this study, we addressed three research questions: First, what are the profiles of mental health, parenting stress, and parenting competence for the parents who participated in the PILI studies? In addition to providing descriptive profiles across the parents, we hypothesized that parents in this study would report significantly higher levels of mental health symptoms and parenting stress compared with the general population and that significant change in mental health symptoms and parenting stress would not result as a function of participation in the PILI. Second, how does parent change in use of intervention strategies relate to parent self-reported (a) symptoms across a variety of mental health categories, (b) parenting stress, and (c) overall feelings of parenting competence? We hypothesized that higher rates of mental health symptoms, increased levels of parenting stress, and lower levels of parenting competence would be related to decreased change in use of the parent intervention strategies. Third, how does child growth in spoken language from pre- to post-intervention relate to parent self-reported (a) symptoms across a variety of mental health categories, (b) parenting stress, and (c) overall feelings of parenting competence? We hypothesized that higher rates of mental health symptoms, increased levels of parenting stress, and lower levels of parenting competence would be related to less growth in child language. To the extent that mental health and stress are related to parent and child intervention-related outcomes as hypothesized, the need for interventions that also target parental mental health and stress (e.g., MBSR; Neece, 2014) would be beneficial to increase the efficacy of PILIs.

## Method

### Participants

Participants in the present analysis were 43 parent–child dyads who participated in either a randomized group design study of the PILI against treatment-as-usual (McDuffie et al., 2018) or a double-blind randomized placebo-controlled trial of PILI plus lovastatin (Thurman et al., 2020). These studies took place between 2015 and 2018. In both studies, the youth participants were between 10 and 17 years of age and were recruited nationally through a list of previous participants who participated in previous studies conducted by this group of researchers and agreed to be recontacted, a university research registry, and social media posts and emails to members of a national advocacy and support organization focused on FXS. The youth participant age range was restricted to 10–17 years due to the use of lovastatin in the clinical trial, with that being the youngest range approved for the drug’s use by the U.S. Food and Drug Administration.

Nineteen parent–child dyads completed the randomized group design study, nine of whom were assigned to a treatment-as-usual waitlist control group and 10 to PILI. The youth were males with FXS and their biological mothers. Twenty-four parent–child dyads were included from the PILI and lovastatin trial, including 22 male youth participants and two females, with caregiver participants being 21 biological mothers, two adoptive mothers, and one adoptive father. Fourteen of the latter dyads were assigned to PILI to parent + placebo to youth with FXS and 10 to PILI to parent + lovastatin to youth with FXS. Four parent–child dyads from the treatment-as-usual waitlist control group also participated in the lovastatin trial (with two of the youth with FXS receiving placebo and two of the youth receiving lovastatin). To reduce redundancy in the data, only data from the treatment-as-usual waitlist control group were used for these four dyads in the current analyses. All families resided in North America, with 19 states and one Canadian province represented. Moreover, the sample comprised mostly White, non-Hispanic/Latinx families from middle to upper income households. Additional participant characteristics are presented in Table 1. This study was approved by the institutional review board at the University of California, Davis. Parents provided informed written consent, and assent was obtained from each youth participant.

### Procedure and Measures

#### Parent–Child Language Samples

All parent–child dyads in the two PILI studies completed three shared storytelling interactions using wordless picture books during the 2 weeks prior to, and following, the intervention. For these interactions, three pairs of books were counterbalanced across dyads and time points. As such, the pairs of books were split across the time points so that one book from each pair was read at the pre-intervention time point and the other at the post-intervention time point. The pairs of books were from the same author and similar in content and style. These books were not used during the intervention period, and the families did not receive any feedback or coaching from the clinician on these books. All books were edited to remove any text, were comparable in length, and were uploaded to the iPads that the families used during the 12-week intervention.

#### Transcription

Video recordings of the parent–child language samples were transcribed by trained research assistants using SALT (Systematic Analysis of Language Transcripts; Miller & Iglesias, 2017) according to procedures outlined in the work of Abbeduto et al. (2020). In these procedures, a primary transcriber completes the first draft of a transcript, which is then reviewed and edited by a second transcriber. Following this, the primary transcriber finalizes the transcript based on the second transcriber’s feedback. The SALT software can automatically generate measures of parent and child language from finalized transcripts, including the two child variables of interest here: number of different words produced (NDW; a measure of lexical diversity) and total number of utterances (i.e., a measure of talkativeness).

#### Coding of Parent–Child Language Samples

Finalized SALT transcripts were coded utterance-by-utterance for parent strategy use (i.e., expansions of child utterances, open-ended questions, and intonation prompts) and parent story–related talking. Completely unintelligible and off-topic child utterances were marked during coding, so that they would be excluded from analyses. Interobserver agreement was conducted as detailed in the works of McDuffie et al. (2018) and Thurman et al. (2020) with all variables of interest having intraclass correlation coefficients above .950.

#### Parent Questionnaire Measures

Three questionnaires were administered to parents. These questionnaires targeted self-report of a wide range of mental health symptoms, including but not limited to those that have previously been shown to be common among *FMR1* premutation carriers (e.g., anxiety and depression), as well as stressors commonly associated with the parenting role and that have been previously shown to be elevated in families of youth with disabilities (e.g., Abbeduto et al., 2004; Hartley et al., 2012).

##### Symptom Checklist-90–Revised (SCL-90-R; Derogatis, 1994).

The Symptom Checklist-90-Revised SCL-90-R (Derogatis, 1994) is a 90-item scale that measures mental health symptoms along multiple dimensions: Somatization (e.g., “Headaches,” “Pains in lower back”), Obsessive–Compulsive (e.g., “Repeated unpleasant thoughts that won’t leave your mind,” “Having to do things very slowly to insure correctness”), Interpersonal Sensitivity (e.g., “Feeling others do not understand you or are unsympathetic,” “Feeling uneasy when people are watching or talking about you”), Depression (e.g., “Blaming yourself for things,” “Feeling hopeless about the future”), anxiety (e.g., “Feeling so restless you couldn’t sit still,” “Thoughts and images of a frightening nature”), Hostility (e.g., “Getting into frequent arguments,” “Feeling easily annoyed or irritated”), Phobic Anxiety (e.g., “Feeling afraid in open spaces or on the streets,” “Feeling nervous when you are left alone”), Paranoid Ideation (e.g., “Having ideas or beliefs that others do not share,” “Feeling that you are watched or talked about by others”), and Psychoticism (e.g., “Other people being aware of your private thoughts,” “The idea that someone else can control your thoughts”). In addition to ratings for each of these dimensions, the measure also provides a Global Severity Index, Positive Symptom Total, and Positive Symptom Distress Index. The normative mean for each dimension is 50, with a standard deviation of 10. Lower scores indicate lower levels of mental health challenges. A *T* score ≥ 63 (equivalent to the 90th percentile) on the Global Severity Index, or two or more scores ≥ 63 on any dimension, suggests clinically significant levels of mental health challenges.

##### Parenting Stress Index—Fourth Edition, Short Form.

The Parenting Stress Index—Fourth Edition, Short Form PSI-4-SF (Abidin, 2012) is a 36-item scale that measures parenting stress in the domains of anxiety, mood, relationships, attachment, and family mental health and functioning. A Total Stress score was computed, as were scores on the following subscales: (a) Parental Distress (e.g., “I find myself giving up more of my life to meet my children’s needs than I ever expected”), (b) Parent–Child Dysfunctional Interaction (e.g., “It takes a long time and it is very hard for my child to get used to new things”), and (c) Difficult Child (e.g., “My child’s behavior is more of a problem than I expected”). *T* scores and percentiles are provided, with lower scores indicating lower levels of parenting stress. Percentile scores of 16–84 are considered to be within the normal range, whereas scores between 85 and 89 are considered high, and scores of 90 or above are considered clinically significant.

##### Parenting Sense of Competence Scale.

The Parenting Sense of Competence Scale PSOC (Gibaud-Wallston & Wandersman, 1978) is a 17-item scale that measures parenting competence along two dimensions: Satisfaction (e.g., “Being a good parent is a reward in itself”) and Efficacy (e.g., “I honestly believe that I have all the skills necessary to be a good parent to my child”). Total raw scores range from 17 to 102, and higher scores on this measure indicate higher levels of parenting competence.

Parent age, years of educational attainment, and cognitive ability were also measured at the pre-intervention assessment visit. Cognitive ability was assessed using the Kaufman Brief Intelligence Test–Second Edition (KBIT-2; Kaufman & Kaufman, 2004), which is a brief measure of verbal and nonverbal intelligence from which a full-scale IQ score can be obtained. These measures were useful for describing the sample and understanding limits on the generalizability of the findings. Relationships between these measures and the other parent and youth intervention-related outcomes were also examined.

### Analysis Plan

Analyses were conducted using IBM SPSS Statistics Version 28. To address potential confounding variables within the data set, prior to running subsequent analyses, we confirmed that there were no baseline differences between the groups in terms of age, nonverbal cognitive level, language level, or autism status for the youth participants, as well as age, IQ, and education level for the parent participants. To address the first research question regarding parent profiles of mental health, parenting stress, and parenting competence, we began by computing a series of one-way analyses of variance (ANOVAs) across the various measures of parent well-being by four levels of treatment condition with post hoc Tukey’s honestly significant difference comparisons with examined differences among the treatment groups. Dependent measures in the ANOVAs were (a) *T* scores from the nine primary symptom dimensions on the SCL-90-R as well as the Global Severity Index, (b) *T* scores from the three subdomains on the PSI-4-SF as well as Total Parenting Stress, and (c) total scores from the PSOC in the four treatment conditions. There were no significant group differences across any of the measures, and thus, the four treatment conditions were collapsed to provide a more robust representation of parent mental health and well-being in this population. For parent report on the SCL-90-R and PSI-4-SF, we conducted a series of one-sample *t* tests against the normed mean of 50 to highlight potential differences in this sample of parents compared with the general population. Last, we conducted a series of paired-samples *t* tests to address potential changes to parent mental health and stress over the course of the intervention. Shapiro–Wilk tests of normality were conducted for the primary variables of interests with most yielding nonsignificant findings, and thus, parametric analyses were used. For the first research question, we hypothesized that (a) parents in this sample would have significantly higher scores across the SCL-90-R symptom dimensions as well as on the PSI-4-SF compared with the normed sample, and (b) parent symptom severity on the SCL-90-R and parenting stress scores would not change as a function of their participation in the PILI.

To address the second and third research questions, descriptive summaries of youth and parent performance during the language intervention were reported. Additionally, for the second research question examining the relationship between measures of mental health and parenting stress and competence with parent change in use of language intervention strategies, as well as the third research question addressing potential carryover effects of parent mental health and well-being on child language outcomes, a series of Pearson correlations were conducted separately across the three intervention groups (e.g., PILI only, PILI + placebo, and PILI + lovastatin). These correlations explored the relationships between the change in parental use of targeted intervention strategies (i.e., open-ended questions, expansions, and intonation prompts) and story-related talking from pre- to post-intervention as well as changes in child language outcomes (i.e., number of different words and story related talking), with clinically significant measures of mental health, parenting stress, and parenting competence described above. Additional parent characteristics (i.e., age, educational attainment, and IQ) were also considered. Again, Shapiro–Wilk tests of normality were conducted for the primary variables of interest, with most yielding nonsignificant findings, and thus, parametric analyses were used. Hypotheses for the second and third research questions were (a) that lower levels of mental health symptom expression and parenting stress as well as higher levels of parenting competence pre-intervention would be related to a greater change in parent-strategy use, and (b) that lower levels of mental health symptom expression and parenting stress as well as higher levels of parenting competence pre-intervention would be related to greater change in child-spoken language.

## Results

### Research Question 1: What Are the Profiles of Mental Health, Parenting Stress, and Parenting Competence for the Parents Who Participated in the PILI Studies?

Table 2 displays descriptive statistics for parent scores on the SCL-90-R and PSI-4-SF as well as the number and percentage of parents who met the instruments’ cutoffs for clinical significance. With regard to the SCL-90-R, of the 43 parents in this sample, 23% scored within the clinically significant range for *Somatization*, 44% for *Obsessive–Compulsive*, 33% for *Interpersonal Sensitivity*, 26% for *Depression*, 19% for both *Anxiety* and *Hostility*, 16% for *Phobic Anxiety*, 9% for *Paranoid Ideation*, and 30% for *Psychoticism*, and 40% had scores within the clinically significant range for their overall global symptom severity (i.e., the *Global Severity Index*). On the PSI-4-SF, 16% had scores within the clinically significant range on the *Parental Distress* domain, 14% on the *Parent–Child Dysfunctional Interaction* domain, 35% on the *Difficult Child* domain, and 16% for *Total Stress*.

Table 2 also displays how the current sample compares with the normative samples used in the development of the SCL-90-R and PSI-4-SF (i.e., whether the mean scores for the current sample were significantly different from a *T* score of 50). Overall, parents in this study had substantially elevated levels of mental health symptoms when compared with the normed sample. In particular, they presented with significantly higher levels of symptoms on the dimensions of *Somatization* (*M* = 54.70, *SD* = 10.25), *t*(42) = 3.01, *p* < .01; *Obsessive–Compulsive* (*M* = 59.28, *SD* = 10.62), *t*(42) = 5.73, *p* < .001; *Interpersonal Sensitivity* (*M* = 57.28, *SD* = 11.07), *t*(42) = 4.31, *p* < .001; *Depression* (*M* = 57.35, *SD* = 9.26), *t*(42) = 5.20, *p* < .001; and *Hostility* (*M* = 53.42, *SD* = 9.53), *t*(42) = 2.35, *p* < .05, as well as on the *Global Severity Index* (*M* = 56.35, *SD* = 11.01), *t*(42) = 3.78, *p* < .001). Cohen’s *d* effect size estimates from these significant differences range from small effects (Hostility, *d* = 0.359) to large effect sizes (Obsessive–Compulsive, *d* = 0.874) as detailed in Table 2. Furthermore, these parents reported significantly higher levels of parenting stress compared with the normed sample for the domains of *Parent–Child Dysfunctional Interaction* (*M* = 53.98, *SD* = 7.96), *t*(42) = 3.28, *p* < .01, and *Difficult Child* (*M* = 58.77, *SD* = 9.68), *t*(42) = 5.94, *p* < .001, as well as in the overall metric, *Total Stress* (*M* = 54.91, *SD* = 7.93), *t*(42) = 4.06, *p* < .001, with Cohen’s *d* effect size estimates ranging from medium effects (Parent–Child Dysfunctional Interaction, *d* = 0.500) to large effect sizes (Difficult Child, *d* = 0.906). On the PSOC, the overall mean score at pre-intervention was 73.74 (*SD* = 11.72). There were no significant differences in the mean scores on any of the parent mental health or stress measures from pre- to post-intervention.

### Research Question 2: How Does Parent Change in Use of Intervention Strategies Relate to Parent Self-Reported (A) Symptoms Across a Variety of Mental Health Categories, (B) Parenting Stress, and (C) Overall Feelings of Parenting Competence?

Descriptive summaries of youth and parent performance during the language intervention are presented in Table 3. Parents who received coaching through PILI showed significant increases in their use of story-related talking and targeted language strategies (i.e., use of open-ended questions, expansions, and intonation prompts) from pre- to-post-intervention (see Table 3). These findings were consistent with those of the McDuffie et al. (2018) and Thurman et al. (2020) studies, which is not surprising given that the present sample is a subset of the participants from those studies. Results from a series of one-way ANOVAs also yielded significant differences in change in strategy use across the three treatment groups in their use of expansions, *F*(2, 31) = 4.70, *p* = .016, and intonation prompts, *F*(2, 31) = 3.37, *p* = .047, with parents in the PILI-only group outperforming those in the PILI + placebo group for expansions and parents in the PILI + lovastatin group outperforming those in the PILI-only group for intonation prompts.

Moreover, the magnitude of change in the use of strategies from pre- to post-intervention was related to several indices of mental health and parenting stress and competence indicating moderate (*r* = .30–49) to strong (*r* = .50–1.00) correlations with varying profiles emerging by treatment group (see Table 4). Parent use of story-related talking was significantly and negatively related to symptoms of *Somatization* (*r* = −.646, *p* < .05) in the PILI-only group as well as to *Interpersonal Sensitivity* (*r* = −.575, *p* < .05), *Depression* (*r* = −.638, *p* < .05), and *Hostility* (*r* = −.645 *p* < .05) in the PILI + lovastatin group. For the measure of parenting stress, the *Parent–Child Dysfunctional Interaction* (*r* = .492, *p* < .05) domain was significantly and positively associated with story-related talking in the PILI + placebo group and *Parenting Sense of Competence* (*r* = .599, *p* < .05) was significantly and positively related to story-related talking for the PILI-only group. Parent use of expansions was significantly and negatively correlated with *Somatization* (*r* = −.625, *p* < .05) and *Depression* (*r* = −.566, *p* < .05). Expansion use was also significantly and positively correlated with *Interpersonal Sensitivity* (*r* = .573, *p* < .05) and *Parenting Sense of Competence* (*r* = .693, *p* < .05) in the PILI-only group and with the *Difficult Child* (*r* = .471, *p* < .05) domain in the PILI + placebo group. Last, for both the PILI-only and PILI + lovastatin groups, parent use of intonation prompts was significantly and negatively correlated with symptoms of *Obsessive–Compulsive* (*r* = −.645, *p* < .05; *r* = −.606, *p* < .05)*, Depression* (*r* = −.795, *p* < .01; *r* = −.775, *p* < .01), and the *Global Severity Index* (*r* = −.688, *p* < .05; *r* = −.615, *p* < .05), respectively, in addition to *Somatization* (*r* = −.727, *p* < .01) for the PILI-only group and *Interpersonal Sensitivity* (*r* = −.571, *p* < .05) and *Hostility* (*r* = −.720, *p* < .01) in the PILI + lovastatin group. The use of intonation prompts was also negatively correlated with *Total Parenting Stress* (*r* = −.770, *p* < .01) and positively correlated with *Parenting Sense of Competence* (*r* = .557, *p* < .05) in the PILI-only group. No significant correlations emerged between parent use of open-ended questions and the measures of mental health symptoms, parenting stress, or parenting competence.

Additional parent characteristics (i.e., parent IQ, age, and education level) were also considered as potential correlates of change in the use of language support strategies. Parent IQ was significantly and positively correlated with change in the use of expansions (*r* = .518, *p* < .05) and intonation prompts (*r* = .614, *p* < .05) in the PILI + placebo group and change in the use of story-related utterances (*r* = .656, *p* < .05) in the PILI + lovastatin group. Parent age was positively related with change in story-related talking (*r* = .561, *p* < .05) in the PILI + lovastatin group and negatively related with change in story-related talking (*r* = −.482, *p* < .05) and the use of open-ended questions (*r* = −.525, *p* < .05) for the PILI + placebo group.

### Research Question 3: How Does Child Growth in Spoken Language From Pre- to Post-Intervention Relate to Parent Self-Reported (A) Symptoms Across a Variety of Mental Health Categories, (B) Parenting Stress, and (C) Overall Feelings of Parenting Competence?

As with parent change in use of strategies, changes across child language outcomes were significantly higher for the youth that participated in PILI regardless of treatment condition compared to the youth who did not receive PILI (see Table 3). Moreover, results of the final research question indicated potential carryover effects of parent mental health and parenting stress and competence on child language outcomes within the strong correlation range (e.g., *r* = .50–1.00) with variability across the different treatment conditions (see Table 5). In particular, symptoms of *Somatization* (*r* = −.836 and −.835; *p* < .01), *Obsessive–Compulsive* (*r* = −.614 and −.674, *p* < .05), *Depression* (*r* = −.697 and −.715, *p* < .05), and the *Global Severity Index* (*r* = −.655 and −.699, *p* < .05), as well as *Parental Distress* (*r* = −.580 and −.691, *p* < .05) on the PSI-4-SF, were all negatively and significantly correlated with changes in child story-related talking and the number of different words, respectively, used by the child from pre- to post-intervention in the PILI-only group*. Parenting Sense of Competence* was also significantly and positively correlated with change in the number of different words used by the child in the PILI-only group (*r* = .641, *p* < .05). Moreover, for the PILI + placebo group, symptoms of *Hostility* were negatively and significantly associated with change in the number of different words used by the child (*r* = −.596, *p* < .05).

## Discussion

This study was designed to examine relationships among parent characteristics, parent use of the strategies taught in the intervention, and child growth in spoken language over the course of a PILI delivered remotely across two studies (McDuffie et al., 2018; Thurman et al., 2020). First, profiles of parent mental health, stress, and competence and the extent to which these parent characteristics changed from pre- to post-intervention for a group of parents who participated in two PILI studies were examined. Second, relationships between parent change in use of strategies taught during the PILI and parent mental health, stress, and competence were examined. Third, relationships between child intervention-related growth in spoken language and parental mental health, stress, and competence were examined. Results from this study provide preliminary support for significant relationships among parent characteristics—including mental health, parenting stress, and parenting competence—and parent- and child-related intervention outcomes. These relationships may explain some of the variability in change in use of parent strategies, as well as growth in child language, across the treatment groups over the course of the intervention. These findings could help guide the development of future interventions for families of children with neurodevelopmental disorders.

### Profiles of Mental Health Symptoms and Parenting Stress

To answer the first research question, mental health symptoms and parenting stress in parents of children with FXS were examined. Information regarding the biological mothers’ genetic status was not collected in the works of McDuffie et al. (2018) and Thurman et al. (2020). However, 40 of the 43 parents in this study were biological mothers of children with FXS. Therefore, they had to be carriers of either the *FMR1* premutation or full mutation, and thus, they were also genetically susceptible to mental health challenges. Consistent with past research, many of these parents had clinically significant levels of mental health symptoms across multiple dimensions on the SCL-90-R compared with the general population (e.g., Gossett et al., 2016; Johnston et al., 2003; Potter et al., 2022). Additionally, these parents reported clinically significant levels of parenting stress on multiple domains of the PSI-4-SF and at higher levels than the normed sample (e.g., McCarthy et al., 2006). These findings suggest that these parents would benefit from services focused on improving their mental health and stress related to parenting.

### Correlates of Parent Strategy Use

#### Mental Health Symptoms and Change in Use of Parent Strategies

Our second research question was to determine how parent change in use of strategies taught during the PILI related to parent mental health, stress, and competence. Although different profiles and patterns emerged across the treatment groups in the relationships between mental health symptoms and parent change in use of intervention strategies, it was consistently found, with only one exception, that when mental health symptoms did relate to strategy use, the associations were negative. Thus, parents who were exhibiting higher levels of mental health symptoms found it more challenging to use responsive strategies during interactions with their child. This finding is consistent with past research in families of both neurotypical children and children with other developmental disabilities (e.g., Justice et al., 2019; Paulson et al., 2009). Moreover, in families of children with FXS, many maternal characteristics have been linked to variability in parental responsivity, including maternal IQ and education, as well as maternal stress and depressive symptoms (Sterling et al., 2013; Wheeler et al., 2007).

These relationships should be further explored in a larger data set to discern more clearly how mental health symptoms should be addressed in order to maximize parent and child treatment gains. Importantly, given the format of parent-implemented interventions and their intent to engage the parent as a proxy for a trained clinician, establishing methods to support the parent, beyond learning the intervention strategies, could prove vital to the ongoing success of these interventions. School-based speech-language pathologists and Individualized Education Program teams should be cognizant of the potential for higher levels of mental health challenges and parenting stress in these families and provide information regarding access to community resources for parents of children with disabilities when deemed clinically appropriate.

#### Parenting Stress and Competence and Change in Use of Parent Strategies

There were significant associations among parenting stress and competence and parent change in use of strategies, with some deviations in the expected direction of these relationships. As hypothesized, when *Parenting Sense of Competence* related to parent change in use of strategies, the relationship was positive (i.e., parents who felt more competent had greater change in their use of strategies). For parenting stress, however, these results were more varied and did not always align with the hypothesis. There was one instance of *Total Parenting Stress* relating negatively to change in use of intonation prompts in one of the treatment groups. In contrast, *Parent–Child Dysfunctional Interaction* and *Difficult Child* related positively to parents’ use of story-related talking and expansions in the PILI-only group. Although these findings are contrary to expectations, it is possible given the challenges that parents were experiencing in trying to engage with the child—and child difficulty in particular—that the parent worked to be more responsive in their approach to the interaction (e.g., providing story models and expanding on child utterances) as opposed to using more directive strategies such as open-ended questions and intonation prompts that are used to elicit a verbal response from the child.

#### Additional Parent Characteristics

In addition to the findings surrounding the relationships between intervention gains and parent mental health and feelings of parenting stress and competence, both parent cognitive ability and age were significantly related to parent change in strategy use. In particular, there was a positive association between cognitive ability and change in use of some strategies in both the PILI + placebo and PILI + lovastatin treatment groups, which is consistent with past research on maternal responsivity in FXS (Sterling et al., 2013). These findings serve as reminders that intervention materials should be designed to be accessible to those of varying ages and cognitive abilities.

### Carryover Effects on Child Outcomes

Our third research question was to determine how child intervention–related gains in spoken language parent mental health, stress, and competence. Past research has consistently demonstrated that parent well-being influences child development in both typical and neurodevelopmental populations (e.g., Warren & Brady, 2007). These effects were also observed in this study. Specifically, various parent mental health symptoms were negatively related to child growth in story-related talking and lexical diversity, although these results varied across treatment groups. Unexpectedly, there were no significant associations between parenting stress and intervention-related changes in child language. Moreover, although parenting sense of competence was only related to lexical diversity in one of the treatment groups, this relationship was in the hypothesized direction. Given that there were limited and unexpected findings in the relationship between parenting stress and parent change in use of strategies, it is possible that parenting stress does not affect parent and child intervention gains to the extent that parent mental health does. Additionally, there are other child intervention–related outcomes that might be more related to parenting stress (e.g., child behavior and engagement), which were not examined in this study. Future work should consider exploring the role that parenting stress plays in parent-implemented interventions and parent–child interactions in a larger sample, especially given the high levels of parenting stress reported by these parents.

### Addressing Parent Well-Being in Parent-Implemented Interventions

Parent-implemented interventions in their current form tend to focus exclusively on improving child outcomes without considering parent or family well-being (Karst & Van Hecke, 2012; Stahmer & Pellecchia, 2015; Wainer et al., 2017). However, given the current results, future parent-implemented interventions may benefit from addressing aspects of parental well-being, including mental health and stress, as a part of the intervention. The time-intensive nature of these interventions is likely to be burdensome for some parents (Pickard et al., 2016; Ruppert et al., 2016), potentially leading to increased stress. Therefore, intentionally focusing on reducing parent stress as a part of the intervention could be beneficial, not only for parent outcomes but also for child well-being (Neece, 2014).

One potential intervention that could be beneficial is MBSR, which is a well-established and empirically supported intervention that has been shown to improve family functioning by reducing parent stress and mental health symptoms and decreasing child behavior problems in families of children with developmental disabilities (Chan & Neece, 2018; Neece et al., 2019). Hunter et al. (2019) investigated the feasibility of using the Headspace Take 10 program, which is a free smartphone app–based program, with biological mothers of children with FXS; they found that many of the mothers were able to complete the 10-day program, and that a majority of those who did, found it to be helpful. Therefore, mindfulness programs that are easy to access and complete may be particularly beneficial for these mothers, especially free and/or low-cost options that can be completed independently from home. Other interventions such as counseling with a psychologist or the use of medications such as selective serotonin receptor reuptake inhibitors (SSRIs) can also be helpful for the anxiety, depression, obsessive–compulsive behavior, and other psychopathology that is common in *FMR1* premutation carriers (Hagerman et al., 2018). Speech-language pathologists working with parents could also consider partnering with mental health professionals (e.g., school social workers or counselors) to assess parent well-being and provide support and resources to families when needed.

### Limitations and Future Directions

This study has several limitations. First, each of the four groups had small sample sizes and, therefore, findings should be considered preliminary in nature. Additionally, as this was a secondary analysis, information was not collected from parents on how their participation in the intervention was influenced by their own feelings of mental health or parenting stress. Another limitation is the relative lack of cultural or economic diversity in the sample. As such, it is not clear how these findings would translate to more diverse communities, including those from historically underserved communities of color and those from lower resourced communities. Last, the sample of parents largely comprised biological mothers, which is consistent with a majority of past studies of families of children with FXS. Therefore, potential similarities and differences in parent characteristics or parent and child intervention–related changes based on parental status (e.g., mother vs. father and biological vs. adoptive parent) could not be explored. Future studies could explore this area since mothers with the *FMR1* premutation are at greater risk for psychopathology (Hagerman et al., 2018). Moreover, given the expansion of digital mental health tools and interventions (Torous et al., 2020), future work should leverage such innovative approaches to implementation sciences by addressing the utility of adding empirically supported digital mental health interventions (Boucher et al., 2021; Petrovic & Gaggioli, 2020) to support the parent or caregiver while not adding undue burden or restricting accessibility or scalability (Schueller & Torous, 2020). Additionally, future studies should target enrollment of fathers in these families, as very little is known about their well-being or the father–child relationship in families of children with FXS (Potter et al., 2022).

In summary, this study is consistent with the vast body of literature supporting the important role that parents play in advocating for and supporting their child’s development across the life span (Landry et al., 2003; Sanders & Turner, 2018). This study highlights not only parents’ ability to acquire and implement strategies commonly used by trained clinicians but also their ability to achieve these gains while experiencing heightened levels of mental health symptoms and stress. As such, it is vital that ongoing research and clinical implementations consider not only the important role that parents play when engaging with their child but also how to support them as individuals beyond their parenting role.

## Figures and Tables

**Table 1.** Participant characteristics.

Pre-intervention child characteristics				
	*M* (*SD*) Range			
Variable	No PILI (*n* = 9 M)	PILI only (*n* = 10 M)	PILI + placebo (*n* = 14 M)	PILI + lovastatin (*n* = 8 M, 2 F)
Age (years)	12.26 (1.13) 10.42–14.08	13.92 (2.26) 10.58–17.17	13.22 (2.69) 10.09–17.77	14.10 (2.42) 10.14–17.28
Nonverbal cognitive ability^a^	41.78 (8.51) 36–62	41.80 (8.72) 36–62	42.71 (5.80) 36–54	45.60 (10.15) 36–62
Expressive vocabulary^b^	5.71 (1.23) 2.83–7.00	6.02 (1.69) 3.75–9.50	5.68 (1.72) 3.25–9.92	6.43 (2.76) 2.58–12.17
Receptive vocabulary^c^	5.84 (1.32) 2.67–6.83	6.48 (2.56) 3.50–12.25	5.71 (1.97) 2.75–10.58	6.05 (2.82) 3.17–11.00
Autism symptom severity^d^	5.22 (1.56) 2–7	6.00 (2.31) 2–10	6.71 (2.16) 2–9	7.11 (2.42) 3–10
Pre-intervention parent characteristics				
	*M* (*SD*) Range			
Variable	No PILI (*n* = 9 BM)	PILI only (*n* = 10 BM)	PILI + placebo (*n* = 13 BM, 1 AF)	PILI + lovastatin (*n* = 8 BM, 2 AM)
Age (years)	43.44 (6.23) 35–54	44.20 (6.00) 36–53	45.14 (5.80) 36–58	44.40 (7.98) 31–59
Cognitive ability^e^	104.00 (17.00) 73–129	109.20 (12.20) 86–133	102.21 (8.87) 87–120	107.60 (15.35) 89–130
Parent education (years)	15.11 (2.42) 12–18	15.30 (1.77) 12–18	15.71 (1.98) 13–18	16.10 (1.85) 13–18

*Note*. PILI = parent-implemented language intervention; M = male; F = female; BM = biological mother; AF = adoptive father; AM = adoptive mother. PPVT-4 = Peabody Picture Vocabulary Test–Fourth Edition; KBIT-2 = Kaufman Brief Intelligence Test–Second Edition.

a IQ on the Leiter International Performance Scale–Revised (Roid & Miller, 1997).

b Age-equivalent score on the Expressive Vocabulary Test–Second Edition (Williams, 2007).

c Age-equivalent score on the PPVT-4 (Dunn & Dunn, 2007).

d Autism severity score on the Autism Diagnostic Observation Schedule–Second Edition (Lord et al., 2012).

e Full-scale IQ on KBIT-2 (Kaufman & Kaufman, 2004).

**Table 2.** Parent-reported mental health symptoms and parenting stress.

Score	Mean T score (*SD*) range	*n* (%) with T score ≥ 63	One-way ANOVA *t* (*p*)	Cohen’s *d* effect size
SCL-90-R dimension scores				
Somatization	54.70 (10.25) 35–78	10 (23)	3.01** **(.004)**	0.458
Obsessive–Compulsive	59.28 (10.62) 37–80	19 (44)	5.73*** **(< .001)**	0.874
Interpersonal Sensitivity	57.28 (11.07) 39–69	14 (33)	4.31*** **(< .001**)	0.658
Depression	57.35 (9.26) 34–74	11 (26)	5.20*** **(< .001)**	0.794
Anxiety	52.44 (10.85) 37–79	8 (19)	1.48 (.148)	0.225
Hostility	53.42 (9.53) 40–74	8 (19)	2.35* **(.023)**	0.359
Phobic Anxiety	51.93 (10.10) 44–80	7 (16)	1.25 (.217)	0.191
Paranoid Ideation	50.21 (9.65) 41 −72	4 (9)	0.14 (.888)	0.188
Psychoticism	52.95 (9.72) 44–74	13 (30)	1.99^+^ (.053)	0.304
Global Severity Index	56.35 (11.01) 32–80	17 (40)	3.78*** **(< .001)**	0.577
PSI-4-SF subscale scores				
Parental Distress	51.09 (9.90) 35–77	7 (16)	0.72 (.473)	0.110
Parent–Child Dysfunctional Interaction	53.98 (7.96) 40–74	6 (14)	3.28** **(.002)**	0.500
Difficult Child	58.77 (9.68) 34–80	15 (35)	5.94*** **(< .001)**	0.906
Total Stress	54.91 (7.93) 35–74	7 (16)	4.06*** **(< .001)**	0.619

*Note*. Cohen’s *d* of 0.20 indicates a small effect size, 0.50 a medium effect size, and 0.80 a large effect size. Significant values are highlighted in bold. ANOVA = analysis of variance; SCL-90-R = Symptom Checklist-90–Revised; PSI-4-SF = Parenting Stress Index–Fourth Edition, Short Form.

+ *p* < .100.

* *p* < .050.

** *p* < .010.

*** *p* < .001.

**Table 3.** Parent uptake of intervention strategies and child language outcomes.

	Mean Δ (*SD*)				One-way ANOVA *F*(*p*)	
Strategy & outcome	No PILI	PILI only	PILI + placebo	PILI + lovastatin	No PILI vs. PILI	PILI
Parent language strategies						
Story-related talking	−2.48 (21.68)	113.47 (41.15)	77.00 (39.87)	105.73 (36.27)	19.77*** **(*p* < .001)**	2.94^+^ (*p* = .068)
Open-ended questions	0.70 (8.51)	34.97 (19.88)	18.40 (14.41)	29.63 (16.16)	9.19*** **(*p* < .001)**	3.12^+^ (*p* = .058)
Expansions	0.30 (3.72)	33.57 (12.78)	18.81 (10.32)	26.83 (12.55)	17.26*** **(*p* < .001)**	4.70* **(*p* = .016)**
Intonation prompts	0.00 (3.27)	19.10 (11.94)	26.71 (22.08)	39.00 (13.69)	10.57*** **(*p* < .001)**	3.37* **(*p* = .047)**
Child language outcomes						
Story-related talking	7.67 (57.64)	87.20 (33.58)	53.19 (54.73)	79.50 (39.58)	5.25** **(*p* = .004)**	1.91 (*p* = .165)
Number of different words	0.33 (30.70)	64.60 (27.42)	38.62 (33.68)	62.50 (35.31)	8.10*** **(*p* < .001)**	2.43 (*p* = .104)

*Note*. All treatment groups significantly increased their use of parent strategies compared to the non-PILI (parent-implemented language intervention) group. With regard to the treatment group comparisons, the PILI-only group outperformed the PILI + placebo group in their use of expansions and the PILI + lovastatin group outperformed the PILI-only group in their use of intonation prompts. Significant values are highlighted in bold. ANOVA = analysis of variance.

+ *p* < .100.

* *p* < .050.

** *p* < .010.

*** *p* < .001.

**Table 4.** Correlates of parent strategy use.

Variable	Story-related talking			Open-ended questions			Expansions			Intonations prompts		
**Symptom Checklist-90–Revised**												
	**TG1**	**TG2**	**TG3**	**TG1**	**TG2**	**TG3**	**TG1**	**TG2**	**TG3**	**TG1**	**TG2**	**TG3**
Somatization	**−.646** *	.175	−.118	−.472	.142	−.109	**−.625** *	−.077	−.409	**−.727** **	−.216	−.165
Obsessive–Compulsive	−.405	.323	−.541	−.187	.412	−.226	−.318	−.072	−.468	**−.645** *	−.149	**−.606** *
Interpersonal Sensitivity	.424	.187	**−.575** *	.515	.097	.027	**.573** *	−.048	−.251	.136	−.318	**−.571** *
Depression	−.526	.239	**−.638** *	−.296	.228	−.256	**−.566** *	.167	−.407	**−.795** **	−.076	**−.775** **
Hostility	−.423	.231	**−.645** *	−.270	−.039	−.159	−.185	−.107	−.392	−.499	−.182	**−.720** **
Global Severity Index	−.470	.268	−.545	−.230	.236	−.067	−.389	−.003	−.366	**−.688** *	−.172	**−.615** *
**Parenting Stress Index-4-SF and Parenting Sense of Competence**												
	**TG1**	**TG2**	**TG3**	**TG1**	**TG2**	**TG3**	**TG1**	**TG2**	**TG3**	**TG1**	**TG2**	**TG3**
P–C Dysfunctional Interaction	−.331	**.492** *	.286	−.277	.355	−.112	−.385	.030	−.266	−.478	.140	.065
Difficult Child	.344	.426	.175	.271	.357	−.267	.252	**.471** *	−.402	−.121	.457	−.073
Total Parenting Stress	−.197	.369	.149	−.052	.315	−.062	−.334	.237	−.315	**−.770** **	.235	−.107
Parenting Sense of Competence	**.599** *	−.064	.148	.505	−.217	−.038	**.693** *	−.052	.465	**.557** *	.136	.393
**Parent characteristics**												
	**TG1**	**TG2**	**TG3**	**TG1**	**TG2**	**TG3**	**TG1**	**TG2**	**TG3**	**TG1**	**TG2**	**TG3**
Parent education	.296	.109	.228	.226	−.026	.250	.302	.276	.512	.462	.072	.129
Parent IQ	.167	.356	**.656** *	.011	.272	.390	.055	**.518** *	−.057	.454	**.614** *	.301
Parent age	−.061	**−.482** *	**.561** *	−.157	**−.525** *	.363	.004	−.423	.266	−.110	−.182	.366

*Note*. TG1 = parent-implemented language intervention (PILI)–only treatment group; TG2 = PILI + placebo treatment group; TG3 = PILI + lovastatin treatment group; P–C = parent–child. Correlation coefficients between .50 and 1 are indicative of a strong correlation, values between .30 and .49 indicate a medium correlation, and values below .29 indicate a small correlation. Significant values are highlighted in bold.

* *p* < .050.

** *p* < .010.

**Table 5.** Correlates of child language outcomes.

Variable	Child story-related talking			No. different words		
**Symptom Checklist-90–Revised**						
	**TG1**	**TG2**	**TG3**	**TG1**	**TG2**	**TG3**
Somatization	**−.836** **	−.306	−.309	**−.835** **	−.060	−.367
Obsessive–Compulsive	**−.614** *	−.334	**−.487** ^+^	**−.674** *	−.034	−.227
Interpersonal Sensitivity	.382	−.444	−.223	.314	−.309	.128
Depression	**−.697** *	−.256	**−.521** ^+^	**−.715** *	−.138	−.117
Hostility	−.492	−.423	**−.454** ^+^	−.455	**−.596** *	−.030
Global Severity Index	**−.655** *	−.345	−.355	**−.699** *	−.145	−.035
**Parenting Stress Index-4-SF and Parenting Sense of Competence**						
	**TG1**	**TG2**	**TG3**	**TG1**	**TG2**	**TG3**
PC–Dysfunctional Interaction	−.293	−.017	−.174	−.345	−.102	−.366
Difficult Child	.381	.394	−.334	.170	.239	−.548^+^
Total Parenting Stress	−.334	.093	−.222	−.532	−.003	−.296
Parenting Sense of Competence	.526	.291	.297	**.641** *	.264	.086
**Child characteristics**						
	**TG1**	**TG2**	**TG3**	**TG1**	**TG2**	**TG3**
Child age	**−.570** *	.012	.101	−.377	.221	.145
Child IQ	.221	.090	−.076	.279	−.007	.392

*Note*. TG1 = PILI-only treatment group; TG2 = PILI + placebo treatment group; TG3 = PILI + lovastatin treatment group; P–C = parent–child. Correlation coefficients between .50 and 1 are indicative of a strong correlation, values between .30 and .49 indicate a medium correlation, and values below .29 indicate a small correlation. Significant values are highlighted in bold.

+ *p* < .100.

* *p* < .050.

** *p* < .010.

## Data Availability

The data sets used and/or analyzed during this study are available from the corresponding author on reasonable request.
